# A Web-Based Application for Risk Stratification and Optimization in Patients With Cardiovascular Disease: Pilot Study

**DOI:** 10.2196/46533

**Published:** 2023-08-03

**Authors:** Avinash Pandey, Marie Michelle D'Souza, Amritanshu Shekhar Pandey, Hassan Mir

**Affiliations:** 1 Department of Medicine University of Ottawa Ottawa, ON Canada; 2 Cambridge Cardiac Care Cambridge, ON Canada; 3 Department of Medicine McMaster University Hamilton, ON Canada; 4 Division of Cardiology and Division of Cardiac Prevention and Rehabilitation University of Ottawa Heart Institute Ottawa, ON Canada

**Keywords:** atherosclerotic cardiovascular disease, guideline-directed medical therapy, mHealth, mobile health, risk stratification, secondary prevention, web application

## Abstract

**Background:**

In addition to aspirin, angiotensin-converting enzyme inhibitors, statins, and lifestyle modification interventions, novel pharmacological agents have been shown to reduce morbidity and mortality in atherosclerotic cardiovascular disease patients, including new antithrombotics, antihyperglycemics, and lipid-modulating therapies. Despite their benefits, the uptake of these guideline-directed therapies remains a challenge. There is a need to develop strategies to support knowledge translation for the uptake of secondary prevention therapies.

**Objective:**

The goal of this study was to test the feasibility and usability of Stratification and Optimization in Patients With Cardiovascular Disease (STOP-CVD), a point-of-care application that was designed to facilitate knowledge translation by providing individualized risk stratification and optimization guidance.

**Methods:**

Using the REACH (Reduction of Atherothrombosis for Continued Health) Registry trial and predictive modeling (which included 67,888 patients), we designed a free web-based secondary risk calculator. Based on demographic and comorbidity profiles, the application was used to predict an individual’s 20-month risk of cardiovascular events and cardiovascular mortality and provides a comparison to an age-matched control with an optimized cardiovascular risk profile to illustrate the modifiable residual risk. Additionally, the application used the patient’s risk profile to provide specific guidance for possible therapeutic interventions based on a novel algorithm. During an initial 3-month adoption phase, 1-time invitations were sent through email and telephone to 240 physicians that refer to a regional cardiovascular clinic. After 3 months, a survey of user experience was sent to all users. Following this, no further marketing of the application was performed. Google Analytics was collected postimplementation from January 2021 to December 2021. These were used to tabulate the total number of distinct users and the total number of monthly uses of the application.

**Results:**

During the 1-year pilot, 47 of the 240 invited clinicians used the application 1573 times, an average of 131 times per month, with sustained usage over time. All 24 postimplementation survey respondents confirmed that the application was functional, easy to use, and useful.

**Conclusions:**

This pilot suggests that the STOP-CVD application is feasible and usable, with high clinician satisfaction. This tool can be easily scaled to support the uptake of guideline-directed medical therapy, which could improve clinical outcomes. Future research will be focused on evaluating the impact of this tool on clinician management and patient outcomes.

## Introduction

Atherosclerotic cardiovascular disease (ASCVD) is a leading cause of morbidity and mortality among adults in North America [1,2]. Historically, guidelines have focused on a 3-pronged approach with antithrombotic therapy, lipid-lowering therapy, and antihypertensive therapy [1]. Despite this, there remains a residual risk for cardiovascular events such as acute coronary syndrome, congestive heart failure, and cardiovascular death [3,4].

Recent landmark trials have resulted in a paradigm shift in the management of patients with ASCVD [2]. The Canadian Cardiovascular Society incorporated these into a recent guideline on evidence-based secondary prevention [2]. In addition to usual antithrombotic therapies, dual pathway inhibition with vascular-dose rivaroxaban is recommended for patients with polyvascular disease (defined as the presence of atherosclerosis in 2 or more arterial beds, including coronary artery disease, cerebrovascular disease, and peripheral arterial disease) [5]. In addition to statin therapy, ezetimibe [6] and proprotein convertase subtilisin/kexin type 9 inhibitors [7,8] are recommended for patients with persistently elevated lipid levels. For those with elevated triglycerides, icosapent ethyl [9] is also recommended. Sodium glucose cotransporter-2 inhibitors [10-12] and glucagon-like peptide-1 agonists [13,14] are also indicated as oral hypoglycemic agents to reduce risk in diabetics with ASCVD. Nonpharmacological interventions, including smoking cessation [15], weight reduction, increased physical activity, dietary changes, and stress and depression management, remain vital [2]. Cardiac rehabilitation referrals are encouraged [16].

Despite the established benefit of guideline-directed medical therapy (GDMT), uptake of these therapies remains a challenge in patients with ASCVD [17-20]. This results in worse clinical outcomes for patients [21]. There is an urgent need to develop strategies to support knowledge translation for the uptake of GDMT.

To support this, our team developed a novel, easy-to-use, point-of-care, provider-facing web-based application. The primary aim of the Stratification and Optimization in Patients With Cardiovascular Disease (STOP-CVD) application was to stratify and optimize patients by incorporating a simplified guideline-based algorithm of care based on patient demographics. The purpose of this quality improvement (QI) project is to demonstrate the feasibility and usability of this tool among physicians providing care for patients with ASCVD.

## Methods

### Application Development

We developed a free, web-based application using PHP: Hypertext Preprocessor. Google Analytics was integrated to track usage. No individual user data was collected or stored. The application was developed to be accessible on desktop and mobile devices running all available operating systems.

### Risk Calculator

The REACH (Reduction of Atherothrombosis for Continued Health) Registry was a large cross-sectional study that included 67,888 patients with ASCVD in 44 countries [22]. Predictive modeling identified key demographic and medical history factors to estimate cardiovascular events and cardiovascular deaths at 20 months [23]. These include age, sex, current residence location, smoking status, body mass index, and 1 or more of the following (current and history of): (1) cardiovascular event in the previous 12 months, (2) atrial fibrillation, (3) polyvascular disease, (4) aspirin therapy, and (5) statin therapy.

Our team leveraged this to create an interactive risk calculator, which was incorporated into the STOP-CVD application. Figure S1 in Multimedia Appendix 1 displays a screenshot of the user interface of the risk calculator. The application additionally displays the risk of cardiovascular events and cardiovascular death as a bar graph (Figure S2 in Multimedia Appendix 2). A hypothetical patient with matched age, gender, and location demographics with no additional risk factors is displayed by the application in order to illustrate a patient’s potential “intervenable risk” to target with optimal risk factor control (for educational purposes).

### Risk Factor Modification Algorithm

We developed an algorithm for the initiation of GDMT based on a review of landmark clinical trials, systematic reviews, and the 2020 Canadian Cardiovascular Society Secondary Prevention guideline update [2]. To aid in visualization and use, the algorithm was displayed graphically in a flowchart in the STOP-CVD application (Figure S3 in Multimedia Appendix 3). This algorithm outlines the foundational therapies recommended for all patients with ASCVD along with novel therapies for risk factor optimization.

Based on an individual patient’s risk factors (identified in the risk calculator portion of the STOP-CVD application), personalized recommendations for additional therapies based on the risk factor modification algorithm are provided by the application. The indication, drug name, dosing (for pharmacological therapies), and summary of the supporting evidence are included (Figure S4 in Multimedia Appendix 4). Citations and links to the primary literature sources are provided as reference material.

### Privacy and Security

No identifiable patient or clinician user information was used or stored in the STOP-CVD application. There were no privacy or security concerns identified for patients or clinicians.

### Recruitment

The STOP-CVD application was implemented from the Cambridge Cardiac Care Center (CCC), a tertiary cardiovascular clinic in Cambridge, Ontario. It was soft-launched on the internet free of charge [24]. All physicians who referred patients to CCC in 2020 were invited to use the STOP-CVD application to help risk stratify and optimize their patients with ASCVD. The specialties of referring physicians included family medicine, internal medicine, emergency medicine, endocrinology, and cardiology. One-time invitations were sent in January 2021 through email or telephone, depending on the preferred means of communication by the referring physician. The website link was also included in all consult notes sent to referring physicians by physicians at CCC. No further marketing or reminders were used.

### Data Collection

Google Analytics was collected postimplementation from January 2021 to December 2021. These were used to tabulate the total number of distinct users and the total number of monthly uses of the application. All physicians who referred patients to CCC in 2020 were invited to complete a QI survey 3 months postintervention if they had used the application. This survey was administered using their preferred means of communication (email or telephone). The target population was those physicians who had used the application to provide feedback on their experience.

**Figure 1:**
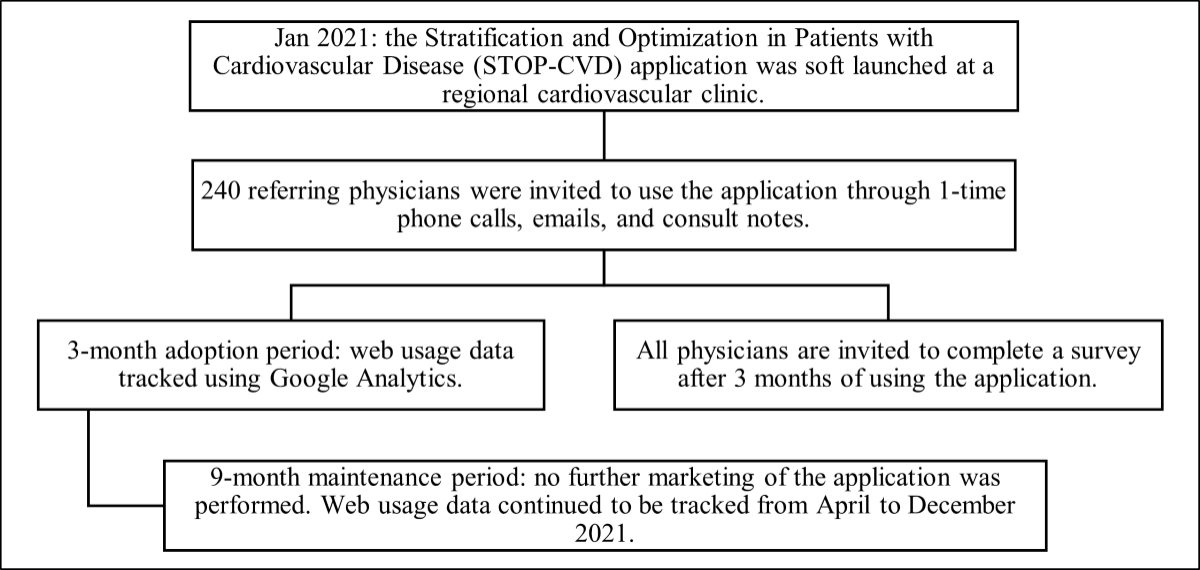
Flow diagram of the STOP-CVD application implementation and evaluation.

All respondents were asked 3 yes-or-no questions: (1) “Is the application functional?” (2) “Is the application easy to use?” (3) “Is the application useful?”. This 3-question survey was designed to answer the “3 measures of usability:” effectiveness, efficiency, and satisfaction, from the seminal book on usability testing, *Usability Evaluation in Industry* [25]. Additional qualitative feedback was solicited from all survey respondents. No participant identifiers were collected. See Figure 1 for details on study recruitment and data collection.

### Evaluation of the Application

The feasibility and usability of this application were evaluated through the RE-AIM (Reach, Effectiveness, Adoption, Implementation, Maintenance) framework [12]. Reach and adoption were assessed based on the number of unique users and frequency of application use within the first 3 months. Implementation was evaluated based on users’ survey responses. Maintenance was assessed based on sustained usage of the application after the initial 3-month adoption phase. During the 9-month maintenance phase, physician users were not contacted further about the application. Effectiveness was not assessed in this study to maintain patient and physician confidentiality.

### Ethical Considerations

The Ottawa Health Science Network Research Ethics Board Research or Quality Improvement checklist was completed. Based on these guidelines, this project was deemed to be a QI project. As such, formal research ethics board approval was deemed not to be necessary and was not obtained.

No identifiable patient or provider data was collected. All aggregate analytic data collected was anonymous. Surveys were anonymous and conducted on an invitation-only basis.

## Results

### Application Usage

The STOP-CVD application was launched on January 1, 2021. A total of 240 physicians were invited to use the application; 47 unique clinicians used the application. Over the year of implementation, the application was used 1573 times, an average of 131 times per month. During the first 3 months (adoption phase), the application was used for a total of 494 patients, an average of 165 patients per month. Peak usage occurred in March 2021, when it was used for 305 patients.

**Figure 2:**
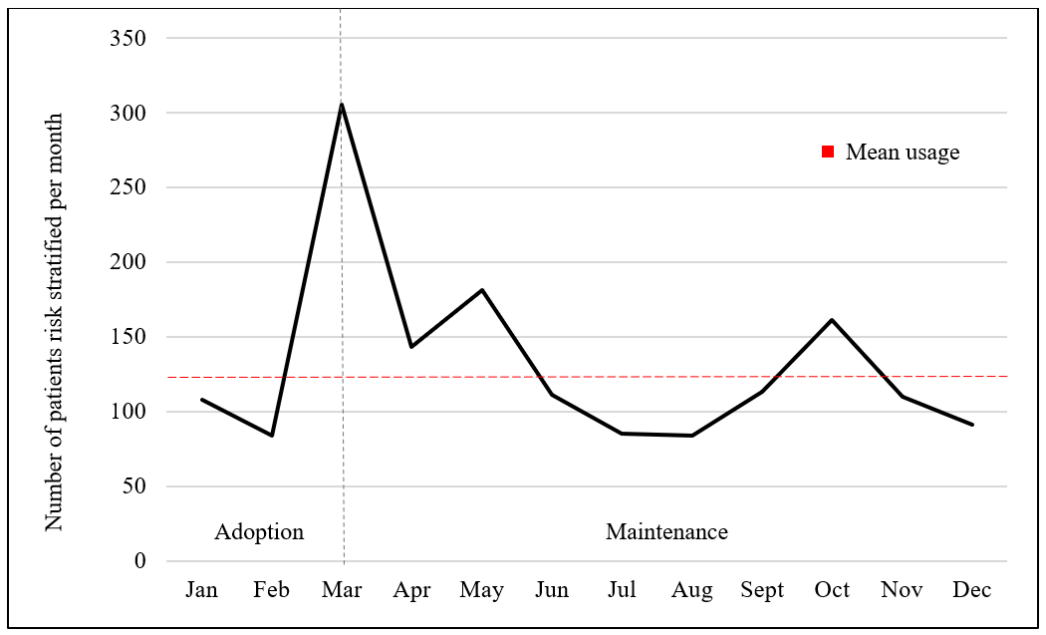
Monthly usage of the Stratification and Optimization in Patients With Cardiovascular Disease (STOP-CVD) application 1 year post implementation.

Over the subsequent 9 months (maintenance phase), the application continued to be used 1079 times, an average of 120 patients per month. See Figure 2 for application usage data.

### User Satisfaction and Feedback

A total of 24 physicians who used the STOP-CVD application agreed to complete the brief postintervention survey. All participants (24/24, 100%) answered “yes” to each of the 3 survey questions: (1) “Is the application functional?” (2) “Is the application easy to use?” (3) “Is the application useful?”

Qualitative feedback was solicited from all survey respondents. A total of 4 users provided qualitative statements. Users stated that the application was “useful for discussions with patients,” “helpful for optimizing patients,” and “similar to a mini cardiology consult.”

One user suggested increased functionality: “It would be helpful for the application to generate a report, which could be added to a patient’s electronic medical record (EMR).” This feedback was implemented after month 3 during the maintenance phase. A printable report function was added to the application (though no patient information was stored in the STOP-CVD application).

## Discussion

### Principal Results

In this QI initiative, we designed and implemented a web-based application that enables evidence-based risk stratification and individualized guidance for the optimization of patients with ASCVD. The STOP-CVD application provides a unique opportunity to use technology to support simplified, algorithm-based knowledge translation of guideline recommendations among clinicians. This pilot project indicates that such a tool is feasible to develop and implement among clinicians. Surveys of clinicians 3 months postimplementation unanimously suggest that the application was highly functional, easy to use, and useful in their clinical practice. Qualitative feedback was obtained and adopted by allowing clinicians to print a report that could be uploaded into the patient’s EMR. These are important findings as they confirm that point-of-care applications aimed at clinicians have the potential to facilitate knowledge translation and use of GDMT, thus improving patient outcomes.

### Comparison With Previous Work

This project seeks to address the significant care gaps among patients with ASCVD. Large studies have demonstrated that patients with known ASCVD are often not prescribed appropriate secondary prevention therapy [26]. Retrospective analysis of 1,489,745 American patients with ASCVD in the National Cardiovascular Data Registry demonstrated that 9% were not prescribed ASA, 20% were not prescribed statins, and 41% were not prescribed angiotensin-converting enzyme inhibitor (ACE-I) or angiotensin receptor blocker (ARB) therapy [26]. Even among patients with ASCVD appropriately prescribed these foundational secondary prevention therapies (antiplatelet therapies, statins, and ACE-I or ARB therapy), the risk of morbidity and mortality remains elevated. A 2020 retrospective cohort study found that patients with postmyocardial infarction (MI) prescribed dual antiplatelet therapy, high-intensity statins, beta-blockers, and ACE-I or ARB therapy had an adjusted hazard ratio of 1.15 for all-cause mortality and 9.56 for hospitalization for MI when compared to the general population [27]. The addition of novel risk factor modification therapies, including antihyperglycemics, lipid optimizing therapies, and dual pathway inhibition, has demonstrated a reduced risk of recurrent cardiovascular events and mortality in multiple landmark clinical trials. Improved uptake of these therapies may improve outcomes among patients with ASCVD [2].

On review of the literature, 3 other tools for secondary risk stratification of patients with established ASCVD were identified: the SMART (Second Manifestations of Arterial Disease) Risk Score [28], SMART-REACH Model [29], and SMART2 Risk Score [30]. Additional tools exist for primary prevention of ASCVD but would not be applicable in this population. Similar to our application, each of these tools used large studies to develop a risk calculator aimed at predicting the risk of future ASCVD events and mortality. However, in addition to a risk calculator, our application provides a simplified algorithm based on risk factors to help clinicians identify what treatments the patient should be initiated on, the indication, dosing, and a summary of available evidence for further reading. This functionality is not provided by other ASCVD risk assessment tools.

There is no literature on the rates of uptake or usage of physician-facing applications for ASCVD secondary prevention or any other form of clinical prediction tool. Historically, physician participation in QI initiatives has been shown to be poor. A cross-sectional survey of Norwegian physicians showed 17% of physicians reported participating in QI during working hours [31]. Among members of the American Board of Family Medicine, 38% of physicians reported participation in a QI activity over the previous year [32]. A total of 20% of participants’ physicians were engaged with the STOP-CVD QI initiative, which appears to be in line with typical physician participation.

Although not assessed in this QI project, previous studies have demonstrated improved GDMT uptake through clinician-facing decision aids. A systematic review of 99 trials found that 70% of physician education interventions improved physician performance metrics [33]. Among studies examining secondary prevention for ASCVD, there is also evidence of the benefit of digital tools for physician education [34]. Vani et al implemented a clinical decision support tool as a part of an EMR and demonstrated an improvement in the prescription of GDMT [34]. Taken together, these studies highlight the potential for using digital tools such as the STOP-CVD application to improve the prescription of GDMT and clinical outcomes.

### Limitations

There are several limitations to this study. First, this is a feasibility and usability study, and therefore no causal conclusions can be derived. Second, no identifying information was obtained, such as clinician demographics or specialty. Due to this, we are unable to perform subgroup analyses of clinicians. Those with a larger roster of patients with ASCVD may use the tool more frequently, but for a smaller proportion of their patients, given comfort with secondary prevention guidelines. Nevertheless, this was a conscious decision to maximize feasibility, usability, and security. Third, this study did not evaluate physician knowledge, prescription practice, or patient outcomes pre- and postintervention. While not the focus of this study, future studies can build on the results of this study.

One limitation to adoption is that this tool currently operates as a web-based application external to EMR. Placing this tool within the EMR workflow as a clinical decision support tool may help to increase adoption (as shown in previous studies) [34]. We chose a web-based application design in order to maximize accessibility to physicians practicing in a variety of clinical environments (including outpatient clinics, which may not use sophisticated EMR systems with clinical decision support capabilities). The application was also optimized for both desktops and mobile phones for ease of use and convenience purposes.

It is important to recognize that volunteer bias and nonresponder bias may affect the validity of our results. A subset of clinicians who were invited (47 of 240) decided to participate in the study, and just over half (24 of 47) completed the postintervention survey. Nonetheless, there was sustained usage and overwhelmingly positive responses by those who completed the postintervention survey.

Despite these limitations, we accomplished the objectives of this study, which were to demonstrate the feasibility and usability of the STOP-CVD application among clinicians from diverse clinical backgrounds providing care for patients with ASCVD. Future research can build on the findings of this study by using rigorous methodology to evaluate the impact of the STOP-CVD tool on clinician knowledge, practice, and patient outcomes.

### Conclusions

Recent landmark trials have shifted the management paradigm for patients with ASCVD. Despite their benefits, the uptake of recommendations remains a challenge. The STOP-CVD application is a novel, point-of-care, easy-to-use, provider-facing web-based application that supports knowledge translation by providing individualized risk factor stratification and optimization guidance. This pilot project indicates that such a tool is feasible to develop and implement among clinicians. This tool can be easily scaled to support the uptake of GDMT, which could improve clinical outcomes. Future research will be focused on the evaluation of the impact of this tool in a randomized, controlled setting. We hope to evaluate its impact on physician and trainee knowledge, prescribing patterns, and patient outcomes.

## Appendix

Multimedia Appendix 1 Screenshot from the Stratification and Optimization in Patients With Cardiovascular Disease (STOP-CVD) application showing the physician-facing REACH (Reduction of Atherothrombosis for Continued Health) risk calculator user interface.

Multimedia Appendix 2 Screenshot from the Stratification and Optimization in Patients With Cardiovascular Disease) STOP-CVD application showing the REACH (Reduction of Atherothrombosis for Continued Health) risk calculator prediction of risk of cardiovascular event and cardiovascular death compared to a patient with the same age, sex, and location demographics without additional risk factors.

Multimedia Appendix 3 Screenshot from the Stratification and Optimization in Patients With Cardiovascular Disease (STOP-CVD) application showing the STOP-CVD secondary prevention algorithm for incorporating guideline-directed medical therapy and lifestyle modification.

Multimedia Appendix 4 Screenshot from the Stratification and Optimization in Patients With Cardiovascular Disease (STOP-CVD) application showing personalized recommendations for additional therapies based on clinical trials and Canadian Cardiovascular Society guidelines with a summary of the available evidence.

## Abbreviations

ACE-I: angiotensin-converting enzyme inhibitors
ARB: angiotensin receptor blocker
ASCVD: atherosclerotic cardiovascular disease
CCC: Cambridge Cardiac Care Center
EMR: electronic medical record
GDMT: guideline-directed medical therapy
MI: myocardial infarction
QI: quality improvement
REACH: Reduction of Atherothrombosis for Continued Health
RE-AIM: Reach, Effectiveness, Adoption, Implementation, Maintenance
SMART: Second Manifestations of Arterial Disease
STOP-CVD: Stratification and Optimization in Patients With Cardiovascular Disease

## References

[1] Mancini GBJ, Gosselin G, Chow B, Kostuk W, Stone J, Yvorchuk KJ, Abramson BL, Cartier R, Huckell V, Tardif JC, Connelly K, Ducas J, Farkouh ME, Gupta M, Juneau M, O'Neill B, Raggi P, Teo K, Verma S, Zimmermann R, Canadian Cardiovascular Society (2014). Canadian cardiovascular society guidelines for the diagnosis and management of stable ischemic heart disease. Can J Cardiol.

[2] Fitchett DH, Leiter LA, Lin P, Pickering J, Welsh R, Stone J, Gregoire J, McFarlane P, Langer A, Gupta A, Goodman SG (2020). Update to evidence-based secondary prevention strategies after acute coronary syndrome. CJC Open.

[3] Dhindsa DS, Sandesara PB, Shapiro MD, Wong ND (2020). The evolving understanding and approach to residual cardiovascular risk management. Front Cardiovasc Med.

[4] Sampson UK, Fazio S, Linton MF (2012). Residual cardiovascular risk despite optimal LDL cholesterol reduction with statins: the evidence, etiology, and therapeutic challenges. Curr Atheroscler Rep.

[5] Eikelboom JW, Connolly SJ, Bosch J, Dagenais GR, Hart RG, Shestakovska O, Diaz R, Alings M, Lonn EM, Anand SS, Widimsky P, Hori M, Avezum A, Piegas LS, Branch KRH, Probstfield J, Bhatt DL, Zhu J, Liang Y, Maggioni AP, Lopez-Jaramillo P, O'Donnell M, Kakkar AK, Fox KAA, Parkhomenko AN, Ertl G, Störk S, Keltai M, Ryden L, Pogosova N, Dans AL, Lanas F, Commerford PJ, Torp-Pedersen C, Guzik TJ, Verhamme PB, Vinereanu D, Kim JH, Tonkin AM, Lewis BS, Felix C, Yusoff K, Steg PG, Metsarinne KP, Bruns NC, Misselwitz F, Chen E, Leong D, Yusuf S, COMPASS Investigators (2017). Rivaroxaban with or without aspirin in stable cardiovascular disease. N Engl J Med.

[6] Cannon CP, Blazing MA, Giugliano RP, McCagg A, White JA, Theroux P, Darius H, Lewis BS, Ophuis TO, Jukema JW, De Ferrari GM, Ruzyllo W, De Lucca P, Im K, Bohula EA, Reist C, Wiviott SD, Tershakovec AM, Musliner TA, Braunwald E, Califf RM, IMPROVE-IT Investigators (2015). Ezetimibe added to statin therapy after acute coronary syndromes. N Engl J Med.

[7] Schwartz GG, Steg PG, Szarek M, Bhatt DL, Bittner VA, Diaz R, Edelberg JM, Goodman SG, Hanotin C, Harrington RA, Jukema JW, Lecorps G, Mahaffey KW, Moryusef A, Pordy R, Quintero K, Roe MT, Sasiela WJ, Tamby JF, Tricoci P, White HD, Zeiher AM, ODYSSEY OUTCOMES Committees and Investigators (2018). Alirocumab and cardiovascular outcomes after acute coronary syndrome. N Engl J Med.

[8] Sabatine MS, Giugliano RP, Keech AC, Honarpour N, Wiviott SD, Murphy SA, Kuder JF, Wang H, Liu T, Wasserman SM, Sever PS, Pedersen TR, FOURIER Steering Committee and Investigators (2017). Evolocumab and clinical outcomes in patients with cardiovascular disease. N Engl J Med.

[9] Bhatt DL, Steg PG, Miller M, Brinton EA, Jacobson TA, Ketchum SB, Doyle RT, Juliano RA, Jiao L, Granowitz C, Tardif JC, Ballantyne CM, REDUCE-IT Investigators (2019). Cardiovascular risk reduction with icosapent ethyl for hypertriglyceridemia. N Engl J Med.

[10] Zinman B, Wanner C, Lachin JM, Fitchett D, Bluhmki E, Hantel S, Mattheus M, Devins T, Johansen OE, Woerle HJ, Broedl UC, Inzucchi SE, EMPA-REG OUTCOME Investigators (2015). Empagliflozin, cardiovascular outcomes, and mortality in type 2 diabetes. N Engl J Med.

[11] Neal B, Perkovic V, Mahaffey KW, de Zeeuw D, Fulcher G, Erondu N, Shaw W, Law G, Desai M, Matthews DR, CANVAS Program Collaborative Group (2017). Canagliflozin and cardiovascular and renal events in type 2 diabetes. N Engl J Med.

[12] Wiviott SD, Raz I, Bonaca MP, Mosenzon O, Kato ET, Cahn A, Silverman MG, Zelniker TA, Kuder JF, Murphy SA, Bhatt DL, Leiter LA, McGuire DK, Wilding JPH, Ruff CT, Gause-Nilsson IAM, Fredriksson M, Johansson PA, Langkilde AM, Sabatine MS, DECLARE–TIMI 58 Investigators (2019). Dapagliflozin and cardiovascular outcomes in type 2 diabetes. N Engl J Med.

[13] Marso SP, Daniels GH, Brown-Frandsen K, Kristensen P, Mann JFE, Nauck MA, Nissen SE, Pocock S, Poulter NR, Ravn LS, Steinberg WM, Stockner M, Zinman B, Bergenstal RM, Buse JB, LEADER Steering Committee, LEADER Trial Investigators (2016). Liraglutide and cardiovascular outcomes in type 2 diabetes. N Engl J Med.

[14] Marso SP, Bain SC, Consoli A, Eliaschewitz FG, Jódar E, Leiter LA, Lingvay I, Rosenstock J, Seufert J, Warren ML, Woo V, Hansen O, Holst AG, Pettersson J, Vilsbøll T, SUSTAIN-6 Investigators (2016). Semaglutide and cardiovascular outcomes in patients with type 2 diabetes. N Engl J Med.

[15] Cahill K, Stevens S, Perera R, Lancaster T (2013). Pharmacological interventions for smoking cessation: an overview and network meta-analysis. Cochrane Database Syst Rev.

[16] Eijsvogels TMH, Maessen MFH, Bakker EA, Meindersma EP, van Gorp N, Pijnenburg N, Thompson PD, Hopman MTE (2020). Association of cardiac rehabilitation with all-cause mortality among patients with cardiovascular disease in the Netherlands. JAMA Netw Open.

[17] Ramsaran E, Preusse P, Sundaresan D, DiMario S, Patel J, Harrison D, Munsell M, Menzin J (2019). Adherence to blood cholesterol treatment guidelines among physicians managing patients with atherosclerotic cardiovascular disease. Am J Cardiol.

[18] Farkouh ME, Boden WE, Bittner V, Muratov V, Hartigan P, Ogdie M, Bertolet M, Mathewkutty S, Teo K, Maron DJ, Sethi SS, Domanski M, Frye RL, Fuster V (2013). Risk factor control for coronary artery disease secondary prevention in large randomized trials. J Am Coll Cardiol.

[19] Rosenson RS, Kent ST, Brown TM, Farkouh ME, Levitan EB, Yun H, Sharma P, Safford MM, Kilgore M, Muntner P, Bittner V (2015). Underutilization of high-intensity statin therapy after hospitalization for coronary heart disease. J Am Coll Cardiol.

[20] Borden WB, Redberg RF, Mushlin AI, Dai D, Kaltenbach LA, Spertus JA (2011). Patterns and intensity of medical therapy in patients undergoing percutaneous coronary intervention. JAMA.

[21] Tully L, Gianos E, Vani A, Guo Y, Balakrishnan R, Schwartzbard A, Slater J, Stein R, Underberg J, Weintraub H, Fisher E, Berger JS (2014). Suboptimal risk factor control in patients undergoing elective coronary or peripheral percutaneous intervention. Am Heart J.

[22] Ohman EM, Bhatt DL, Steg PG, Goto S, Hirsch AT, Liau CS, Mas JL, Richard AJ, Röther J, Wilson PWF, REACH Registry Investigators (2006). The Reduction of Atherothrombosis for Continued Health (REACH) registry: an international, prospective, observational investigation in subjects at risk for atherothrombotic events-study design. Am Heart J.

[23] Wilson PWF, D'Agostino R, Bhatt DL, Eagle K, Pencina MJ, Smith SC, Alberts MJ, Dallongeville J, Goto S, Hirsch AT, Liau CS, Ohman EM, Röther J, Reid C, Mas JL, Steg PG, REACH Registry (2012). An international model to predict recurrent cardiovascular disease. Am J Med.

[24] Residual Cardiovascular Risk: Assessment and Management Guide.

[25] Brooke J, Jordan PW, Thomas B, McClelland IL, Weerdmeester B (1996). SUS: a "quick and dirty" usability scale. Usability Evaluation in Industry, 1st Edition.

[26] Maddox TM, Ho PM, Roe M, Dai D, Tsai TT, Rumsfeld JS (2010). Utilization of secondary prevention therapies in patients with nonobstructive coronary artery disease identified during cardiac catheterization. Circ Cardiovasc Qual Outcomes.

[27] Brown TM, Bittner V, Colantonio LD, Deng L, Farkouh ME, Limdi N, Monda KL, Rosenson RS, Serban MC, Somaratne RM, Zhao H, Woodward M, Muntner P (2020). Residual risk for coronary heart disease events and mortality despite intensive medical management after myocardial infarction. J Clin Lipidol.

[28] Dorresteijn JAN, Visseren FLJ, Wassink AMJ, Gondrie MJA, Steyerberg EW, Ridker PM, Cook NR, van der Graaf Y, SMART Study Group (2013). Development and validation of a prediction rule for recurrent vascular events based on a cohort study of patients with arterial disease: the SMART risk score. Heart.

[29] Kaasenbrood L, Bhatt DL, Dorresteijn JAN, Wilson PWF, D'Agostino RB, Massaro JM, van der Graaf Y, Cramer MJM, Kappelle LJ, de Borst GJ, Steg PG, Visseren FLJ (2018). Estimated life expectancy without recurrent cardiovascular events in patients with vascular disease: The SMART-REACH model. J Am Heart Assoc.

[30] Hageman SHJ, McKay AJ, Ueda P, Gunn LH, Jernberg T, Hagström E, Bhatt DL, Steg PG, Läll K, Mägi R, Gynnild MN, Ellekjær H, Saltvedt I, Tuñón J, Mahíllo I, Aceña Á, Kaminski K, Chlabicz M, Sawicka E, Tillman T, McEvoy JW, Di Angelantonio E, Graham I, De Bacquer D, Ray KK, Dorresteijn JAN, Visseren FLJ (2022). Estimation of recurrent atherosclerotic cardiovascular event risk in patients with established cardiovascular disease: the updated SMART2 algorithm. Eur Heart J.

[31] Deilkås ET, Rosta J, Baathe F, Søfteland E, Lexberg ÅS, Røise O, Rø KI (2022). Physician participation in quality improvement work- interest and opportunity: a cross-sectional survey. BMC Prim Care.

[32] Peterson LE, Jaén CR, Phillips RL (2013). Family physician participation in quality improvement. J Am Board Fam Med.

[33] David A, Davis MD (1995). Changing physician performance: a systematic review of the effect of continuing medical education strategies. JAMA.

[34] Vani A, Kan K, Iturrate E, Levy-Lambert D, Smilowitz NR, Saxena A, Radford MJ, Gianos E (2022). Leveraging clinical decision support tools to improve guideline-directed medical therapy in patients with atherosclerotic cardiovascular disease at hospital discharge. Cardiol J.

