# Supplementary material for: A Web-Based Application for Risk Stratification and Optimization in Patients With Cardiovascular Disease: Pilot Study
Source: JMIR Cardio. 2023 Aug 3;7:e46533. doi: 10.2196/46533 (PMC10436122; doi:10.2196/46533)
Supplement: Multimedia Appendix 2 [file cardio_v7i1e46533_app2.docx]

**Figure S2.** Screenshot from the STOP-CVD application showing REACH risk calculator prediction of risk of cardiovascular event and cardiovascular death compared to a patient with the same age, sex and location demographics without additional risk factors.

**
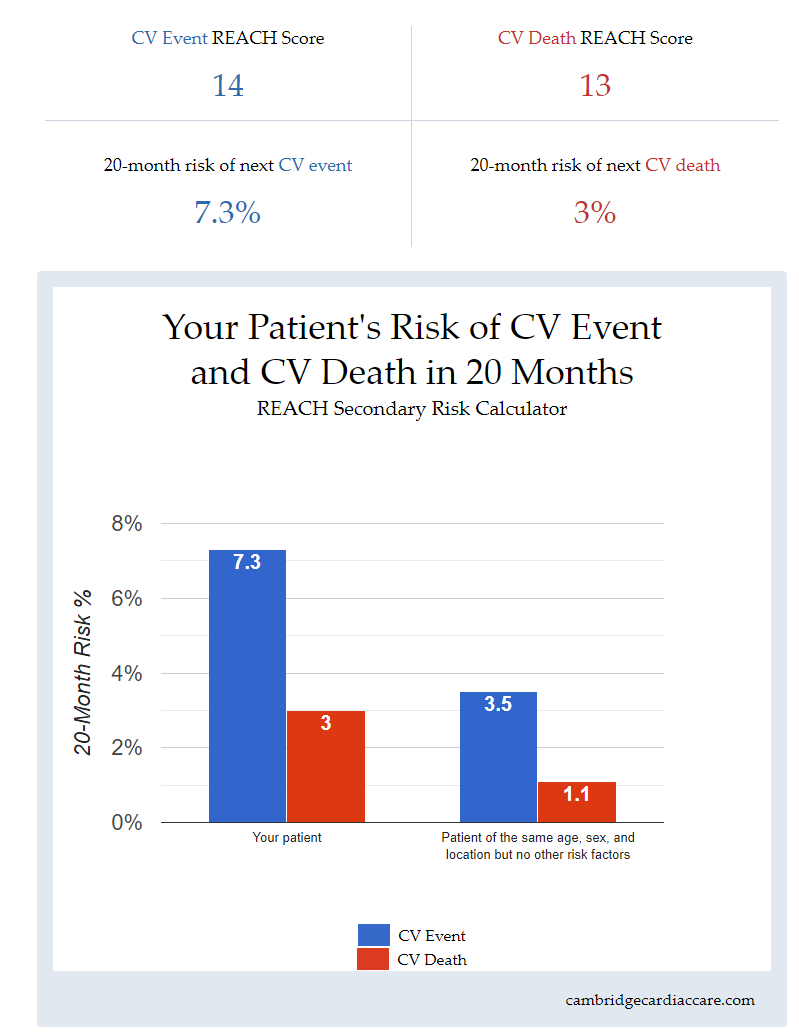
**
