# Supplementary material for: A Web-Based Application for Risk Stratification and Optimization in Patients With Cardiovascular Disease: Pilot Study
Source: JMIR Cardio. 2023 Aug 3;7:e46533. doi: 10.2196/46533 (PMC10436122; doi:10.2196/46533)

**Figure S3.** Screenshot from the STOP-CVD application showing the STOP-CVD secondary prevention algorithm for incorporating GDMT & lifestyle modification.


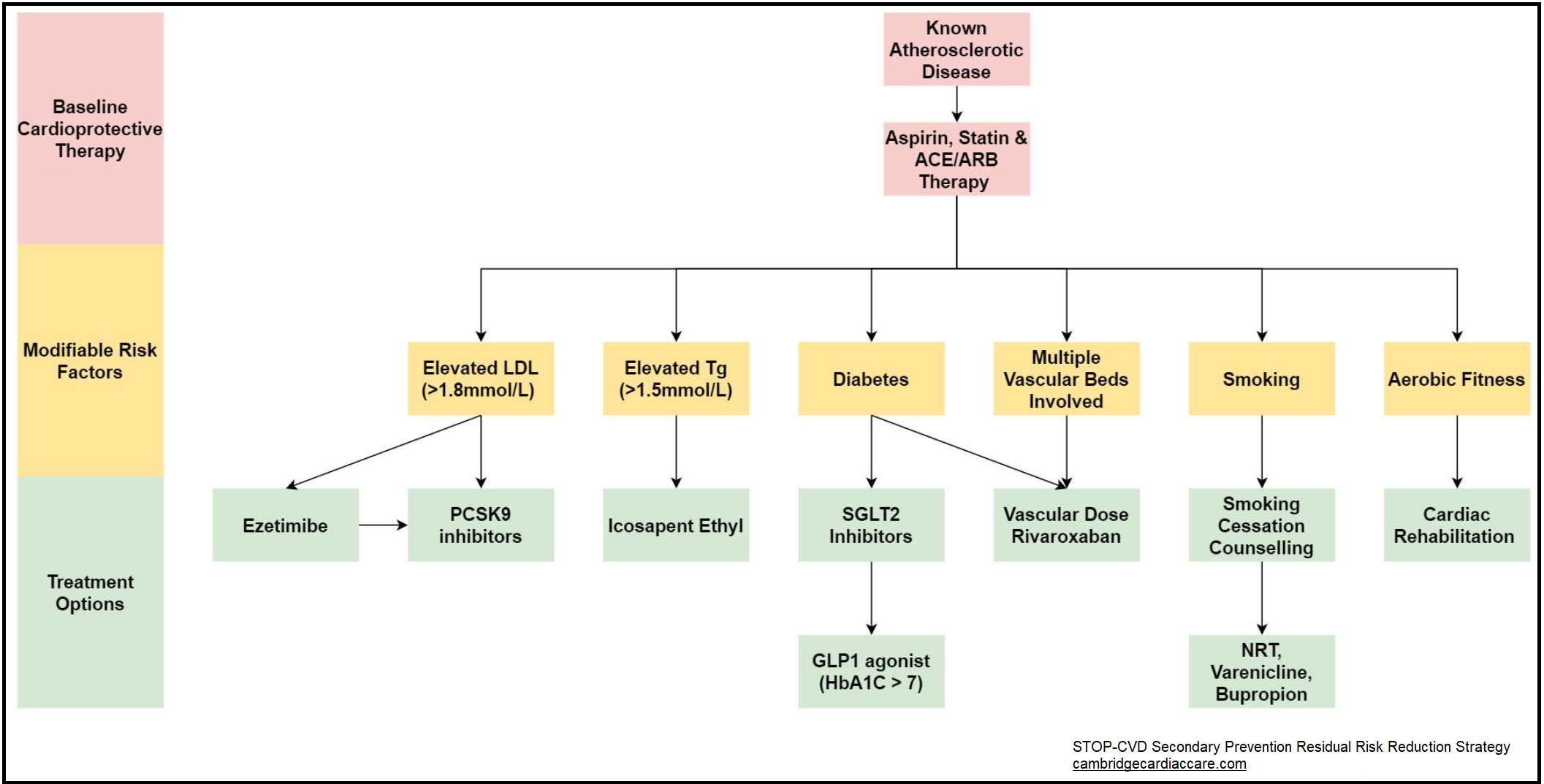

Supplement: Multimedia Appendix 3 [file cardio_v7i1e46533_app3.docx]
