# Supplementary material for: A Web-Based Application for Risk Stratification and Optimization in Patients With Cardiovascular Disease: Pilot Study
Source: JMIR Cardio. 2023 Aug 3;7:e46533. doi: 10.2196/46533 (PMC10436122; doi:10.2196/46533)
Supplement: Multimedia Appendix 4 [file cardio_v7i1e46533_app4.docx]

**Figure S4.** Screenshot from the STOP-CVD application showing personalized recommendations for with additional therapies based on clinical trials and CCS guidelines with a summary of the available evidence.

**
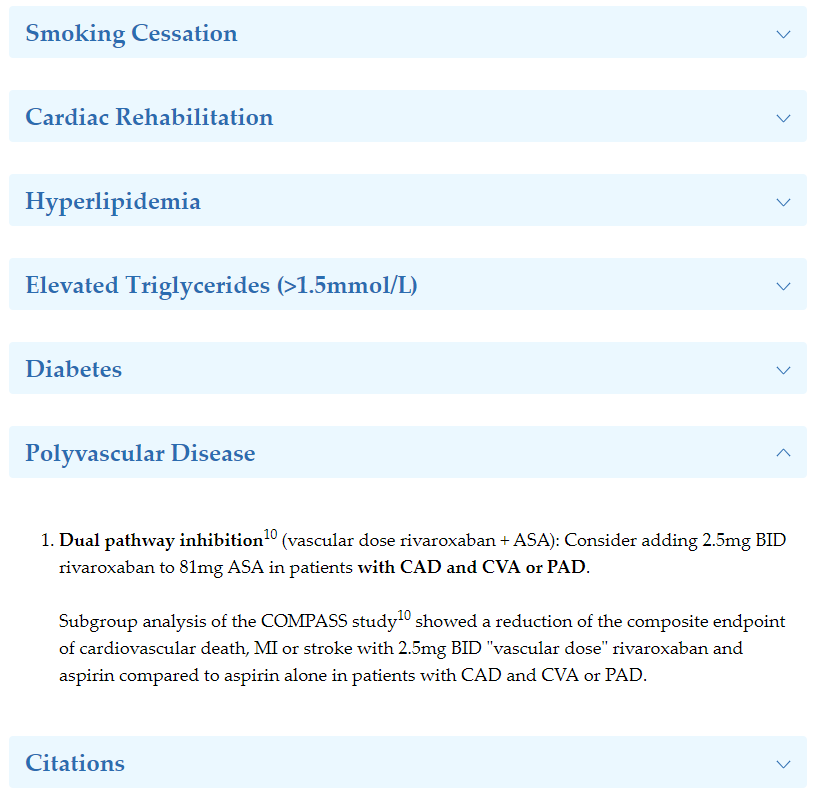
**
